# Acetylation–ubiquitination crosstalk in NLRP3–gasdermin pyroptosis: a substrate-state framework with relevance to ulcerative colitis

**DOI:** 10.3389/fimmu.2026.1976894

**Published:** 2026-10-06

**Authors:** Yuanyang Zhao, Xiaojie Zhu, Enhui Lu, Hai Meng

**Affiliations:** Department of Gastroenterology, Binhai County People’s Hospital, Yancheng, China

**Keywords:** acetylation, GSDMD, NLRP3, pyroptosis, ubiquitination, ulcerative colitis

## Abstract

Acetylation and ubiquitination regulate inflammatory cell death, but evidence that they act together in ulcerative colitis (UC) remains limited. This review examines their interactions in nucleotide-binding oligomerisation domain-, leucine-rich repeat- and pyrin domain-containing protein 3 (NLRP3) and gasdermin D (GSDMD), drawing on a targeted PubMed search for literature published through 10 August 2026. We distinguish coupling tested within one study from separate mechanisms affecting the same substrate, and report human, intestinal and non-intestinal evidence separately. Studies of phosphoglycerate dehydrogenase, the glucocorticoid-induced tumour necrosis factor receptor and histone deacetylase 4 support acetylation–ubiquitination coupling in non-intestinal systems. These findings differ in site resolution and do not establish competition between the modifications at one lysine. Other studies show that acetylation affects NLRP3 assembly or GSDMD activity, while ubiquitination regulates substrate abundance, assembly and degradation. In experimental colitis, selected NLRP3 ubiquitin pathways and GSDMD acetylation mechanisms influence inflammation, but the complete coupled pathways have not been demonstrated in human UC. We organise these findings around four substrate properties: abundance, activation competence, localisation and persistence. This framework is a descriptive synthesis, not a validated evidence hierarchy or clinical biomarker. Its relevance to UC depends on establishing modification coupling in defined intestinal cell types and distinguishing that coupling from broader anti-inflammatory or barrier-protective effects.

## 1 Introduction

Ulcerative colitis (UC) is a chronic inflammatory disease in which epithelial barrier dysfunction and mucosal immune responses interact (1, 2). Pyroptosis can contribute to epithelial injury and inflammatory cytokine release, but the biological consequence depends on the affected cell and the stage of the death pathway.

Inflammasomes activate inflammatory caspases, which process cytokines and cleave gasdermins (3, 4). Gasdermin D (GSDMD) pores permit interleukin (IL)-1β and IL-18 release. Subsequent plasma membrane rupture involves nerve injury-induced protein 1 (NINJ1), as shown in mouse macrophages (5). Pore formation and rupture are separable: GSDMD pores can release IL-1β from living, hyperactivated macrophages (6). Increased pathway-protein expression therefore does not by itself demonstrate pyroptotic death.

Post-translational modifications (PTMs) regulate several steps in this pathway. Acetylation can alter protein interactions and assembly, while ubiquitination can promote activation, change localisation or direct degradation. The outcome depends on the substrate and modification site. This review focuses on nucleotide-binding oligomerisation domain-, leucine-rich repeat- and pyrin domain-containing protein 3 (NLRP3) and GSDMD, for which both modifications have been investigated. The central question is whether acetylation and ubiquitination are experimentally coupled or instead affect the same protein in separate studies. We address that question for each substrate before assessing the relevance to UC.

## 2 Scope and interpretation of the evidence

We conducted a targeted narrative synthesis. PubMed was searched on 22 August 2026 for literature published through 10 August 2026, combining terms for UC or inflammatory bowel disease (IBD), pyroptosis or inflammasomes, NLRP3 or gasdermins, acetylation, ubiquitination and their regulatory enzymes. Additional searches and citation chaining identified mechanistic and intestinal studies. Main-text selection prioritised studies that directly informed substrate modification, coupling or interpretation in UC. **Supplementary Table S1** records the search strategy and a detailed evidence matrix. This was not a systematic review and did not include a formal risk-of-bias assessment.

Three relationships are distinguished in plain language throughout the text: coupling supported by perturbation within one study; convergence of separately tested mechanisms on the same substrate; and single-modification or contextual evidence. The corresponding author-defined labels D1, D2 and D3 are retained in **Supplementary Table S1** for audit purposes. They do not rank study quality. Evidence source is reported independently as human UC tissue, patient-derived experiments, intestinal or colitis models, or non-intestinal systems. The supplementary U1–U4 labels describe these sources; a study can contribute to more than one category. Human expression associations and mixed-IBD cohorts are identified explicitly.

We use four substrate properties to organise the mechanisms: abundance, the amount of protein available; activation competence, its capacity to assemble or execute the pathway; localisation, its access to signalling sites or membranes; and persistence, the balance between continued availability and degradation. These properties are not mutually exclusive and are not validated clinical measurements. **Figure 1** relates them to inflammasome activation and gasdermin-mediated execution. **Table 1** summarises the main mechanistic relationships; **Supplementary Table S1** retains site, model and assay limitations. In site notation, K denotes lysine; R and Q substitutions replace it with arginine and glutamine. RING denotes really interesting new gene, BRCA breast cancer susceptibility protein, and OTU ovarian tumour; these terms occur in conventional protein-family names.

**Figure 1:**
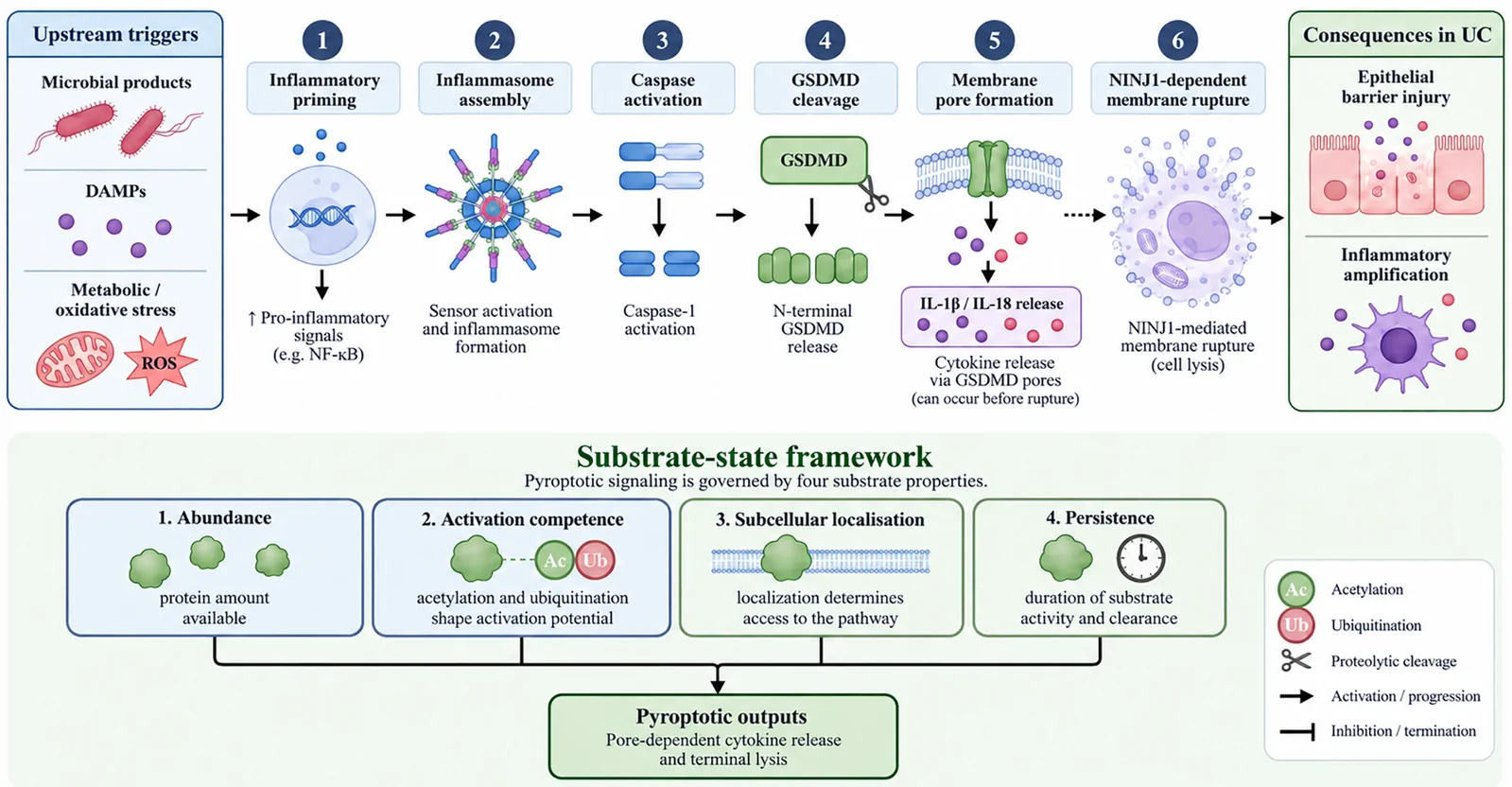
Pyroptotic steps and the substrate-state framework. The upper scheme separates NLRP3 inflammasome assembly from GSDMD cleavage, pore formation and NINJ1-dependent plasma membrane rupture. Cytokine release can occur through pores before terminal rupture. The lower scheme defines abundance, activation competence, subcellular localisation and persistence as overlapping properties for organising modification effects. It is a conceptual framework, not a validated clinical model. NLRP3, nucleotide-binding oligomerisation domain-, leucine-rich repeat- and pyrin domain-containing protein 3; GSDMD, gasdermin D; NINJ1, nerve injury-induced protein 1; IL, interleukin; DAMPs, damage-associated molecular patterns; ROS, reactive oxygen species; NF-κB, nuclear factor kappa B; Ac, acetylation; Ub, ubiquitination.

**Table 1:** Coupled and convergent substrate mechanisms.

Mechanism	Supported relationship	Model and UC boundary
PHGDH–SIRT1/SIRT3; NLRP3/ASC (35)	Same-study coupling: deacetylation accompanies increased ubiquitination and degradation.	Primary mouse macrophages, HEK293T cells and systemic LPS. Combined-site and assay limitations; no human UC validation.
GITR–MARCH7–SIRT2; NLRP3 (36)	Enzyme-level coupling plus a separate branch reducing NLRP3 ubiquitination.	Primary mouse macrophages, HEK293T cells and sepsis models; human observations concern sepsis.
HDAC4; GSDMD K248 (44)	Same-study coupling with K27-linked ubiquitination; acceptor lysine unresolved.	HEK293T/THP-1 cells and LPS mouse inflammation; complete pathway not demonstrated in UC.
NLRP3 acetylation and ubiquitin routes (7, 8, 12, 37–39)	Cross-study convergence on assembly, stability and autophagic clearance.	Non-intestinal mechanisms plus colitis experiments and human UC/mixed-IBD associations; no tested acetylation-dependent route selection.
GSDMD acetylation and clearance (45–48)	Separate site effects and protein-degradation pathways; no demonstrated route selection.	Cellular/non-intestinal studies; K43 mechanism includes DSS colitis. No unified human UC crosstalk pathway.

Coupling means that perturbation links the modifications within one study; it does not imply same-lysine competition. Detailed sites, model provenance, abbreviations and author-defined D/U categories are given in **Supplementary Table S1**. SIRT, sirtuin; NLRP3, nucleotide-binding oligomerisation domain-, leucine-rich repeat- and pyrin domain-containing protein 3; ASC, apoptosis-associated speck-like protein containing a caspase activation and recruitment domain; PHGDH, phosphoglycerate dehydrogenase; GITR, glucocorticoid-induced tumour necrosis factor receptor; MARCH7, membrane-associated RING-CH-type finger 7; HDAC4, histone deacetylase 4; GSDMD, gasdermin D; LPS, lipopolysaccharide; DSS, dextran sodium sulphate; IBD, inflammatory bowel disease; UC, ulcerative colitis; HEK293T, human embryonic kidney 293T cells; THP-1, human monocytic cell line.

## 3 NLRP3 regulation by acetylation and ubiquitination

### 3.1 Acetylation and inflammasome assembly

Sirtuin 2 (SIRT2) and lysine acetyltransferase 5 (KAT5) studies identify distinct acetylation-sensitive features of NLRP3. In macrophages and mouse models of ageing-associated inflammation and insulin resistance, SIRT2-dependent deacetylation restrained inflammasome activation. Single K21R and K22R substitutions reduced activation, whereas K24R alone did not show the same inhibitory phenotype (7). A separate study in macrophages, reconstitution systems and mouse inflammatory models linked KAT5 to acetylation of mouse NLRP3 K24, corresponding to human K26. K24R impaired oligomerisation while retaining recruitment to the dispersed trans-Golgi network (8). Thus, localisation and assembly can be regulated separately. These results should not be combined into a shared KAT5–SIRT2 switch at K24.

Histone deacetylase 9 (HDAC9) had a different reported effect in myeloid models of vascular inflammation: NLRP3 deacetylation accompanied increased inflammasome activation (9). The different models and modification sites prevent a simple activating or inhibitory rule for NLRP3 acetylation. None of these studies established its site-specific mechanism in human UC.

Broader metabolic studies define the upstream context in which these substrate effects occur. Macrophage histone deacetylase 3 (HDAC3) reconfigured mitochondrial fatty-acid oxidation and interleukin-1β production, whereas nicotinamide adenine dinucleotide metabolism supported inflammatory activity in human and mouse intestinal tissue (10, 11). These studies do not establish acetylation–ubiquitination coupling on NLRP3, but they show why cellular metabolism and intestinal compartment must be specified when interpreting an acetylation-dependent phenotype.

### 3.2 Ubiquitin-dependent assembly and degradation

Ubiquitination affects both NLRP3 abundance and assembly. In macrophage and reconstitution experiments, tripartite motif-containing protein 31 (TRIM31) promoted K48-linked ubiquitination and proteasomal degradation of NLRP3 (12). Ariadne RING-between-RING E3 ubiquitin protein ligase 2 (ARIH2) added K48- and K63-linked chains and restrained inflammasome assembly, but ARIH2-deficient human THP-1 cells did not show a corresponding increase in NLRP3 or the adaptor apoptosis-associated speck-like protein containing a caspase activation and recruitment domain (ASC) (13). Chain linkage alone therefore does not establish degradation. The TRIM31 study also illustrates a disease-level limitation: its loss increased selected inflammasome responses yet ameliorated dextran sodium sulphate (DSS) colitis in mice (12).

Ubiquitin-dependent control also acts through ligase exclusion, partner degradation and deubiquitinase complexes. YAP reduced beta-transducin repeat-containing protein 1 (β-TrCP1)-associated K27-linked ubiquitination of NLRP3, whereas A20 promoted K48-linked ubiquitination and degradation of the NLRP3 partner NEK7 (14, 15). Ubiquitin-specific peptidases 7 and 47 supported post-transcriptional assembly, UAF1 complexes stabilised NLRP3 or reinforced priming, and ubiquitin-specific peptidase 16 stabilised NLRP3 in keratinocytes (16–18). These findings identify additional routes controlling abundance or assembly; none demonstrated an acetylation-selected route in intestinal cells.

Sequential ubiquitin editing can further complicate interpretation. In macrophage and reconstitution systems, ring finger protein 125 (RNF125) installed K63-linked chains that enabled recognition by the E3 ligase Cbl-b and subsequent K48-linked ubiquitination at NLRP3 K496, promoting degradation (19). Conversely, deubiquitination can support assembly. BRCA1/BRCA2-containing complex subunit 3 (BRCC3), a deubiquitinase, was required for NLRP3 activation in cellular inflammasome systems (20). Recruitment through its adaptor ABRAXAS2 (also known as ABRO1) supported removal of K63-linked chains, NLRP3–ASC association and ASC oligomerisation in macrophage and reconstitution experiments (21). These studies explain why neither total ubiquitination nor deubiquitinase activity predicts the direction of inflammasome output.

Other E3 ligases illustrate non-degradative roles for ubiquitin in inflammasome competence. Pellino2 acted during priming and required its forkhead-associated and RING-like domains to support NLRP3 ubiquitination and activation, while HUWE1-dependent K27-linked ubiquitination promoted assembly of NLRP3, AIM2 and NLRC4 inflammasomes (22, 23). These mechanisms broaden the ubiquitin-code context but were not tested for dependence on NLRP3 acetylation.

Binding of an acetylation enzyme to ubiquitinated material is not sufficient evidence of modification coupling. Histone deacetylase 6 (HDAC6) transported inflammasome components to an aggresome-like assembly site in macrophage and reconstitution experiments; rescue by catalytically inactive HDAC6 supported a scaffold function (24). In THP-1 cells, pharmacological HDAC6 inhibition mainly affected priming, with no equivalent inhibition when tubastatin A was added after priming (25). These findings concern transport or timing rather than demonstrated acetylation–ubiquitination coupling on NLRP3.

Additional ubiquitin mechanisms reinforce the need to interpret substrate, linkage and destination together. Linear ubiquitin chains supported NLRP3-mediated pyroptosis, a mycobacterial protein redirected NLRP3 ubiquitination, and ubiquitin-specific peptidases 22 and 19 restrained NLRP3 through autophagy-related routes (26–29). Ubiquitin-specific peptidase 48 and OTU deubiquitinase 4 stabilised gasdermin E in cancer models, while RNF31 and TRIM40 linked ubiquitin signalling to colitis inflammation or barrier dysfunction (30–33). These studies are retained as adjacent or disease-context evidence rather than direct proof of acetylation–ubiquitination coupling.

NLRP3 K287 neddylation provides a further adjacent-modification example: it stabilised NLRP3 and opposed TRIM31-dependent degradative ubiquitination in experimental colitis, but did not test acetylation–ubiquitination interdependence (34).

### 3.3 Same-study coupling

Phosphoglycerate dehydrogenase (PHGDH) provides a metabolic link between the modifications. In primary mouse macrophages, human embryonic kidney 293T (HEK293T) reconstitution and lipopolysaccharide (LPS)-induced systemic inflammation, PHGDH inhibition or loss increased oxidised nicotinamide adenine dinucleotide (NAD+) availability and the activity of sirtuins 1 and 3 (SIRT1/SIRT3). NLRP3 and ASC acetylation decreased, while ubiquitination and degradation increased (35). Combined K21/K22/K24 substitutions and SIRT1 perturbation supported acetylation-dependent regulation of protein stability. Some modification measurements used colocalisation, and the combined substitutions do not identify native occupancy or competition at an individual lysine. A separate histone-acetylation branch affected Toll-like receptor 4 (TLR4) transcription, distinguishing priming from protein-stability effects.

A second study linked the glucocorticoid-induced tumour necrosis factor receptor (GITR) to two regulatory branches in primary mouse macrophages, HEK293T cells and sepsis-related mouse models (36). GITR competed with NLRP3 for membrane-associated RING-CH-type finger 7 (MARCH7), reducing NLRP3 K48-linked ubiquitination and autolysosomal degradation. Separately, MARCH7 promoted SIRT2 K48-linked ubiquitination and degradation, increasing NLRP3 acetylation. The latter branch links ubiquitination of an acetylation regulator to modification of NLRP3; the reduction in NLRP3 ubiquitination should not be attributed solely to SIRT2 loss. Human observations in this study concerned sepsis.

The PHGDH and GITR studies support coupling at different regulatory levels, with different assay resolution. Neither establishes same-lysine competition or the complete mechanism in human UC. They provide the direct-coupling component of **Figure 2**; the remaining NLRP3 mechanisms in that figure were tested separately.

**Figure 2:**
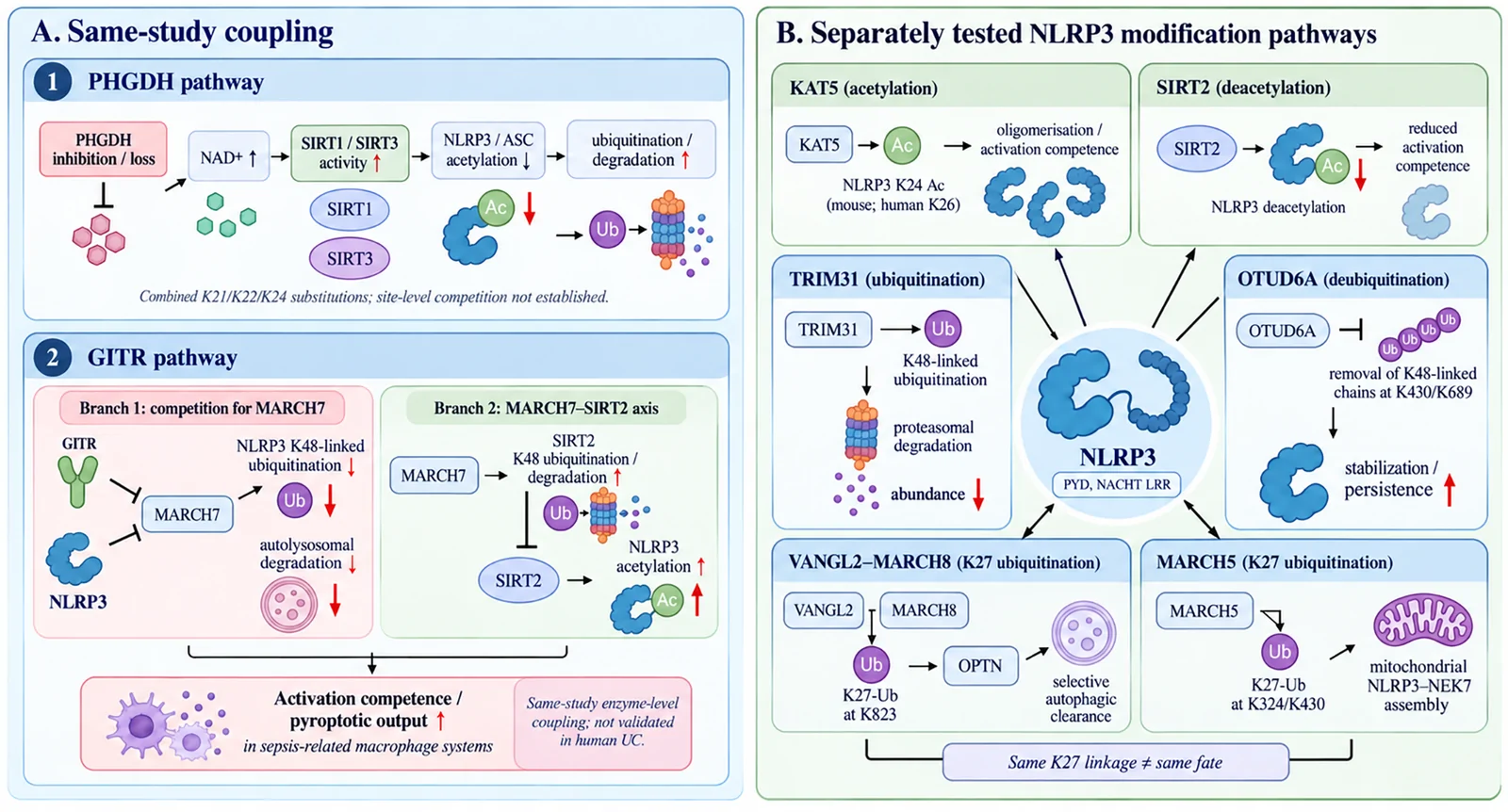
NLRP3 same-study coupling and separately tested modification pathways. **(A)** Same-study coupling: PHGDH inhibition links reduced NLRP3/ASC acetylation to increased ubiquitination and degradation; combined-site substitutions and some colocalisation readouts limit site-level inference. GITR has two distinct branches: reduced MARCH7-dependent NLRP3 ubiquitination, and MARCH7-dependent SIRT2 degradation with increased NLRP3 acetylation. **(B)** Separate studies identify acetylation-sensitive assembly and ubiquitin-dependent stability or routing. Juxtaposition does not imply that acetylation selects a ubiquitin route. Model labels describe the experiments supporting each mechanism. Ac, acetylation; Ub, ubiquitination; PHGDH, phosphoglycerate dehydrogenase; ASC, apoptosis-associated speck-like protein containing a caspase activation and recruitment domain; SIRT, sirtuin; GITR, glucocorticoid-induced tumour necrosis factor receptor; MARCH, membrane-associated RING-CH-type finger; KAT5, lysine acetyltransferase 5; OTUD6A, OTU deubiquitinase 6A; VANGL2, Van Gogh-like protein 2; OPTN, optineurin; NEK7, never in mitosis A-related kinase 7. NLRP3, nucleotide-binding oligomerisation domain-, leucine-rich repeat- and pyrin domain-containing protein 3; LPS, lipopolysaccharide; HEK293T, human embryonic kidney 293T cells; UC, ulcerative colitis; TRIM31, tripartite motif-containing protein 31; NAD+, oxidised nicotinamide adenine dinucleotide.

### 3.4 NLRP3 ubiquitin pathways relevant to colitis

Two studies provide a closer intestinal connection. The OTU deubiquitinase 6A (OTUD6A) study included UC transcriptomic observations and colon tissue from 10 patients with UC and 10 cancer-adjacent controls (37). These human comparisons were associative. Macrophage, reconstitution and mouse experiments supported removal of K48-linked chains at NLRP3 K430/K689, increased sensor stability and a contribution to DSS or 2,4,6-trinitrobenzenesulphonic acid (TNBS) colitis and colitis-associated cancer. The control tissue source and experimental origin of the site-level findings limit interpretation as a human UC mechanism.

Van Gogh-like protein 2 (VANGL2) was examined in mixed-IBD colon tissue and patient-derived blood-cell experiments, as well as myeloid and mouse models (38). VANGL2 recruited membrane-associated RING-CH-type finger 8 (MARCH8) to install K27-linked chains at NLRP3 K823, enabling optineurin (OPTN)-dependent autophagic degradation. Myeloid VANGL2 loss worsened DSS colitis. The human cohort included both UC and Crohn’s disease, so the observations are not UC-only validation. In separate non-intestinal macrophage experiments, membrane-associated RING-CH-type finger 5 (MARCH5)-dependent K27 chains at K324/K430 supported mitochondrial NLRP3 assembly with never in mitosis A-related kinase 7 (NEK7) (39). The contrasting fates show that acceptor site, ligase, reader and location matter alongside linkage.

These intestinal ubiquitin pathways and the acetylation studies support convergence on NLRP3 abundance, assembly and persistence. They do not show that acetylation selects the OTUD6A, MARCH8 or MARCH5 route. The distinction matters for UC: an experimentally supported ubiquitin mechanism in colitis cannot be treated as evidence for a complete acetylation–ubiquitination pathway.

## 4 GSDMD regulation at the execution stage

Gasdermin cleavage exposes a pore-forming fragment whose membrane association and oligomerisation govern execution, as established in cellular and biochemical studies (40–43). Acetylation and ubiquitination can affect different properties of this protein pool. **Figure 3** separates the coupled histone deacetylase 4 (HDAC4) pathway from individual acetylation and degradation mechanisms.

**Figure 3:**
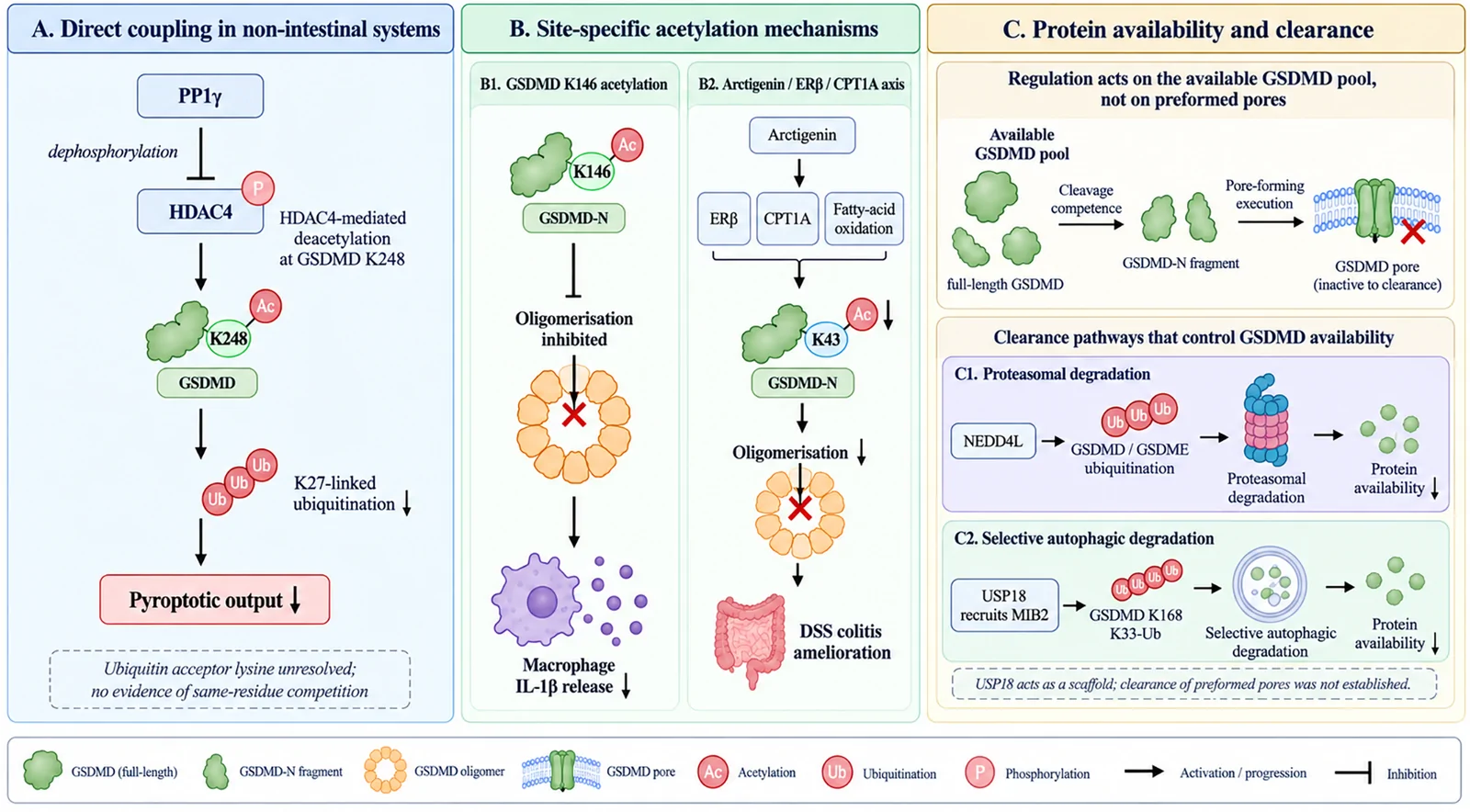
GSDMD acetylation and protein clearance. **(A)** In non-intestinal cell and mouse systems, HDAC4-mediated deacetylation at K248 reduced K27-linked ubiquitination and pyroptotic output. The ubiquitin acceptor lysine remains unresolved; same-residue competition is not established. **(B)** Separate studies identified inhibitory K146 acetylation and an arctigenin-associated K43 mechanism in experimental colitis. **(C)** NEDD4L-mediated proteasomal degradation and USP18–MIB2-mediated autophagic degradation regulate protein availability. These routes were not shown to be selected by acetylation or to remove preformed pores. Ac, acetylation; Ub, ubiquitination; HDAC4, histone deacetylase 4; NEDD4L, neural precursor cell expressed developmentally down-regulated protein 4-like; USP18, ubiquitin-specific peptidase 18; MIB2, mind bomb E3 ubiquitin protein ligase 2. GSDMD, gasdermin D; LPS, lipopolysaccharide; DSS, dextran sodium sulphate; THP-1, human monocytic cell line; PP1γ, protein phosphatase 1 gamma; ERβ, estrogen receptor beta; CPT1A, carnitine palmitoyltransferase 1A; GSDME, gasdermin E; GSDMD-N, amino-terminal GSDMD fragment. K denotes lysine.

### 4.1 Site-specific acetylation and the HDAC4 pathway

In HEK293T and THP-1 cells and an LPS-induced mouse inflammatory model, GSDMD K248 acetylation promoted pyroptotic output, while HDAC4-mediated deacetylation reduced K27-linked ubiquitination and pyroptosis (44). Protein phosphatase 1 gamma (PP1γ) diminished the inhibitory action of HDAC4 through phosphorylation-dependent regulation. This supports coupling between acetylation and ubiquitination, but the ubiquitin acceptor lysine was unresolved. It neither demonstrates competition at K248 nor establishes direct control of cleaved-fragment oligomerisation by this site. The functional PP1γ finding should not be generalised to all PP1 isoforms.

A distinct site had the opposite effect in macrophage and cellular experiments. Increased intracellular D-amino acids promoted GSDMD K146 acetylation through a pyruvate dehydrogenase and acetyl-coenzyme A (acetyl-CoA)-dependent mechanism, inhibiting oligomerisation and reducing IL-1β release (45). Together, the K248 and K146 findings show that acetylation effects must be reported by site and endpoint. Whether the sites are modified in the same protein pool or at different stages remains unresolved; neither complete mechanism has been demonstrated in human UC.

The intestinal acetylation evidence includes arctigenin. In THP-1 cells and DSS-treated wild-type and estrogen receptor beta (ERβ)-deficient mice, arctigenin reduced carnitine palmitoyltransferase 1A (CPT1A)-associated fatty-acid oxidation, acetylation of the GSDMD amino-terminal fragment (GSDMD-N), and oligomerisation (46). K43R and K43Q experiments supported involvement of K43. The acetylation enzyme and any connection to ubiquitination remained unresolved. This is an intestinal single-modification mechanism, and its metabolic direction should not be extrapolated to other GSDMD sites.

### 4.2 Protein availability and ubiquitin-dependent clearance

Neural precursor cell expressed developmentally down-regulated protein 4-like (NEDD4L) promoted proteasomal degradation of GSDMD and gasdermin E (GSDME) in cellular and non-intestinal tissue models (47). Reported GSDMD acceptor sites were K51/K203/K204, with K11/K29/K63 linkages; GSDME involved different sites and linkages, detailed in **Supplementary Table S1**. NEDD4L loss increased gasdermin abundance and susceptibility to cell death. These findings concern the available protein pool, and their intestinal function or dependence on acetylation was not tested.

Ubiquitin-specific peptidase 18 (USP18) acted independently of its catalytic activity as a scaffold recruiting mind bomb E3 ubiquitin protein ligase 2 (MIB2). In macrophage and non-intestinal inflammatory systems, this promoted K33-linked ubiquitination at GSDMD K168 and selective autophagic degradation (48). It should not be described as direct GSDMD deubiquitination or removal of preformed membrane pores. No experiment has shown that K146 or K248 acetylation selects either the NEDD4L or USP18–MIB2 clearance route.

Adjacent gasdermin modifications help define the relevant substrate pool and temporal window. Palmitoylation can regulate membrane localisation and pore formation, whereas broader syntheses catalogue additional post-translational controls of gasdermin D (49–51). These mechanisms are useful comparators for experimental design, but they remain distinct from acetylation–ubiquitination coupling unless modification interdependence is tested on the same substrate pool.

Tripartite motif-containing protein 21 (TRIM21) provides a further distinction between ligase identity and mechanism. Macrophage and cellular experiments linked TRIM21 to GSDMD stability and aggregation, with functional observations in LPS inflammation and DSS colitis (52). Although the study included an *in vitro* ubiquitination assay, domain analysis implicated the carboxy-terminal GSDMD-binding region, and the catalytic RING domain was not required for the principal effects. A specific ubiquitin linkage should therefore not be assigned as the cause of aggregation. Its colitis relevance does not establish acetylation–ubiquitination coupling.

## 5 What the evidence establishes in ulcerative colitis

### 5.1 Human observations and experimental intestinal mechanisms

Human UC studies support involvement of epithelial inflammatory death pathways, but they interrogate different mechanisms. Studies combining patient tissue observations with cell and mouse experiments implicated epithelial mitochondrial fission and PANoptosis, which integrates pyroptosis, apoptosis and necroptosis (53). Another study implicated mammalian sterile 20-like kinase 1 (MST1) and Yes-associated protein (YAP)/p73 signalling in epithelial caspase-1 regulation (54). Human observations also accompanied colonocyte thioredoxin-interacting protein (TXNIP)–NLRP3 signalling and GSDME-associated epithelial injury studies (55, 56). These findings establish disease relevance for selected components; they do not show paired acetylation and ubiquitination of NLRP3 or a gasdermin in human tissue.

The OTUD6A and VANGL2 studies in Section 3.4 provide human observations alongside experimentally tested NLRP3 ubiquitin mechanisms. The arctigenin and TRIM21 studies in Section 4 provide intestinal evidence concerning GSDMD. Their contributions are complementary but cannot be joined into a single causal pathway. **Figure 4** distinguishes human tissue associations, patient-derived experiments, experimental intestinal or colitis models and non-intestinal mechanistic systems without ranking these sources as a hierarchy of quality.

**Figure 4:**
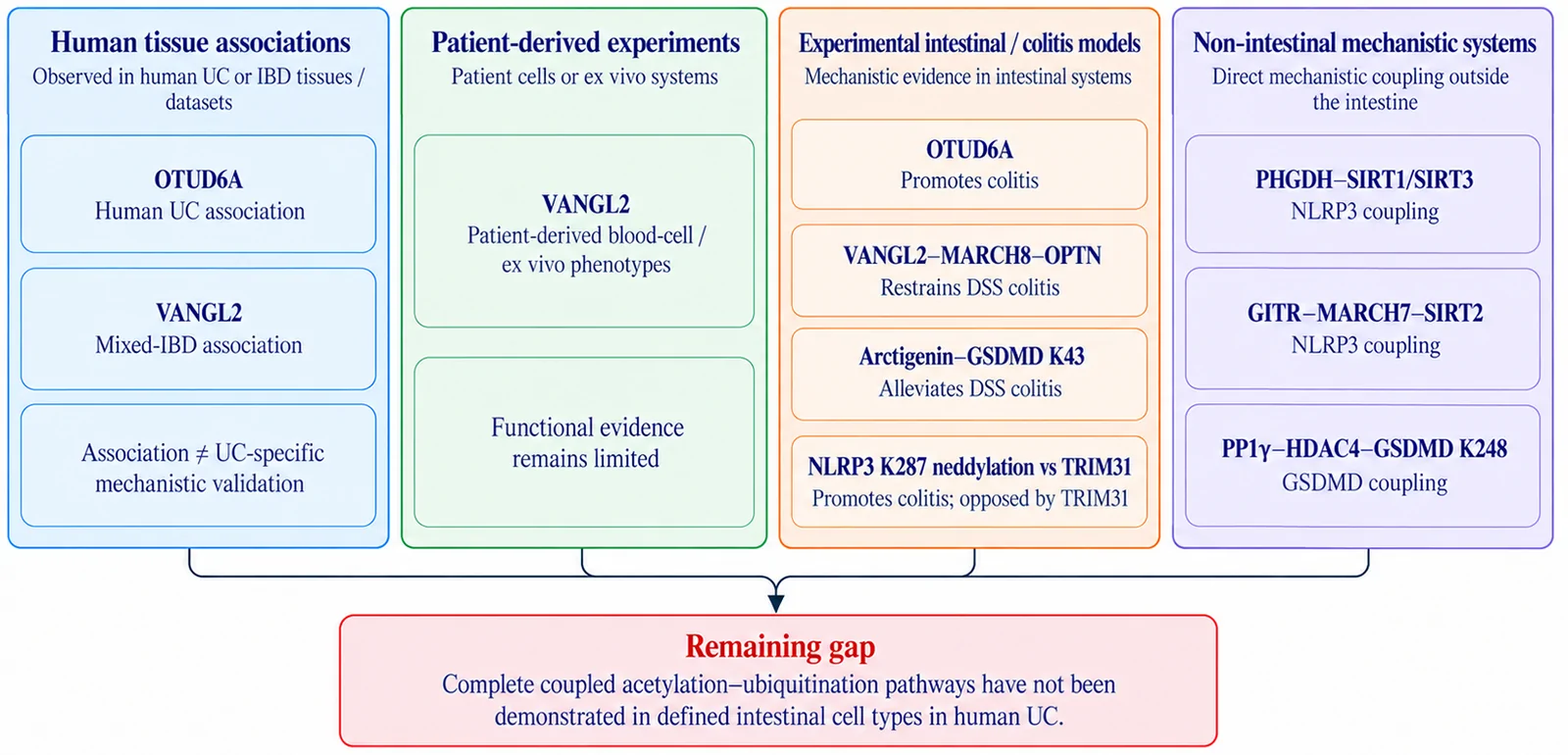
Evidence sources and the remaining link to human UC. Four parallel panels distinguish human tissue associations, patient-derived experiments, experimental intestinal or colitis models and non-intestinal mechanistic systems. These are sources of evidence rather than a hierarchy of quality. The VANGL2 human cohort included both UC and Crohn’s disease, and the OTUD6A controls were cancer-adjacent tissue. A study can contribute observations to several panels. The lower statement identifies the unresolved question: whether the complete coupled pathways operate in defined intestinal cell types in human UC. UC, ulcerative colitis; IBD, inflammatory bowel disease; OTUD6A, OTU deubiquitinase 6A; VANGL2, Van Gogh-like protein 2; PHGDH, phosphoglycerate dehydrogenase; GITR, glucocorticoid-induced tumour necrosis factor receptor; HDAC4, histone deacetylase 4; GSDMD, gasdermin D. SIRT, sirtuin; MARCH, membrane-associated RING-CH-type finger; NLRP3, nucleotide-binding oligomerisation domain-, leucine-rich repeat- and pyrin domain-containing protein 3; DSS, dextran sodium sulphate; OPTN, optineurin; TRIM31, tripartite motif-containing protein 31; PP1γ, protein phosphatase 1 gamma. K denotes lysine.

Additional intestinal studies establish disease context without supplying the missing paired-modification evidence. The phosphoglycerate mutase family member 5 (PGAM5)–NEK7 interaction and a triggering receptor expressed on myeloid cells 1 (TREM1)–ITCH–transforming growth factor beta-activated kinase 1 (TAK1)–caspase-8 axis affected inflammasome or pyroptotic responses in colitis, including an association with anti-tumour-necrosis-factor nonresponse (57, 58). Microbial activation of NAIP/NLRC4 or AIM2, neutrophil SIRT6–hypoxia-inducible factor 1 alpha (HIF-1α) metabolism and ubiquitin-dependent accumulation of stimulator of interferon genes (STING) further illustrate epithelial–myeloid and microbial amplification routes (59–62). Their inclusion preserves the broader UC landscape while keeping the mechanistic claim limited to the pathway tested in each study.

### 5.2 Epithelial protection is not equivalent to modification coupling

Acetylation can affect epithelial integrity outside the NLRP3–GSDMD pathway. In epithelial and experimental-colitis models, SIRT1 supported junctional maintenance through beta-transducin repeat-containing protein 1 (β-TrCP1) and Snail1 (63). Metformin reduced acetyl-CoA availability, signal transducer and activator of transcription 3 (STAT3) acetylation and epithelial inflammation in DSS colitis (64). These studies connect acetylation to barrier function, but do not establish that protection depended on paired modifications of NLRP3 or GSDMD. They are retained as context for interpreting epithelial outcomes, rather than as additional crosstalk mechanisms.

The evidence gap is therefore specific: the complete coupled pathways described in Sections 3.3 and 4.1 have not been demonstrated in human UC. Tissue-level expression can also reflect changing cell composition, and mixed-IBD cohorts cannot establish UC specificity. A UC-relevant mechanism requires the modification relationship, substrate and affected cell type to be identified together. Non-intestinal perturbation studies provide mechanistic support, while intestinal studies determine whether that mechanism operates in the disease setting.

## 6 Therapeutic implications and priorities for validation

The therapeutic question is whether a proposed intervention engages acetylation–ubiquitination coupling, a single modification, or the broader death pathway. Arctigenin is relevant because it links GSDMD acetylation to experimental colitis, although the ubiquitin arm remains untested. Direct sensor or pore inhibition provides a separate therapeutic rationale and cannot by itself validate crosstalk. **Table 2** summarises representative interventions and the inference each supports.

**Table 2:** Representative interventions and their evidence limits.

Example	Evidence and endpoint	What remains unestablished
Arctigenin (46)	THP-1 cells and DSS mice; GSDMD-N K43 acetylation and oligomerisation.	Acetylation enzyme, ubiquitin coupling and clinical efficacy.
PEITC/butyrate (67, 68)	Intestinal cell lines and DSS mice; inflammasome signalling or barrier protection.	Paired acetylation–ubiquitination of NLRP3/GSDMD; efficacy in relapsing human UC.
SRT2104 (69)	31 UC patients enrolled; 26 evaluable; two active doses, no placebo. Limited clinical activity at eight weeks.	Comparative efficacy and engagement of a defined substrate-modification mechanism.
4-Octyl-itaconate liposome (75)	Intravenous delivery to inflamed colon and reduced GSDME-associated epithelial pyroptosis in experimental colitis.	Dual-modification engagement and human treatment efficacy.

These examples distinguish single-modification, pathway-level and clinical evidence; none validates an acetylation–ubiquitination therapy in UC. PEITC, phenethyl isothiocyanate; GSDMD-N, gasdermin D amino-terminal fragment; GSDME, gasdermin E; DSS, dextran sodium sulphate; UC, ulcerative colitis; THP-1, human monocytic cell line.

Direct pathway intervention provides complementary proof of principle. Necrosulfonamide inhibited gasdermin D pore formation in systemic inflammatory models, and pharmacological inhibition of NLRP3 deubiquitination suppressed NLRP3-associated inflammation (65, 66). These approaches support the tractability of the executioner or ubiquitin-regulatory step, but neither demonstrates a colon-specific acetylation–ubiquitination mechanism.

In the human colorectal cell line HT29 and DSS-treated mice, phenethyl isothiocyanate (PEITC) bound TXNIP, disrupted its interaction with NLRP3 and reduced downstream signalling (67). Both dosing before DSS exposure and an early schedule after DSS initiation were studied. These findings concern acute experimental colitis, not established relapsing human UC or modification coupling. Butyrate improved barrier-related outcomes in the human intestinal epithelial cell line Caco-2 BBe and DSS mice through histone deacetylase 8 (HDAC8)-associated regulation of nuclear factor kappa B (NF-κB) and solute carrier family 26 member 3 (SLC26A3), accompanied by an observational human faecal comparison (68). Its barrier effects also do not establish paired substrate modification.

Clinical evidence for sirtuin modulation is limited. An exploratory SRT2104 study enrolled 31 patients with mild to moderate UC and randomised them between two active doses without a placebo group (69). Among 26 evaluable participants, three achieved endoscopic remission and four clinical remission after eight weeks. Colonic exposure was demonstrated, but comparative efficacy was not established. Because substrate modification states were not measured, limited clinical activity cannot be attributed to failure to engage a specific acetylation–ubiquitination mechanism.

A resveratrol derivative, SH-707, suppressed NLRP3 activation through a sirtuin 1-dependent pathway in experimental systems (70). Other metabolic interventions acted more broadly: aryl hydrocarbon receptor–ornithine decarboxylase 1 signalling increased polyamine biosynthesis and limited macrophage pyroptosis, while engineered lactic-acid-producing Saccharomyces cerevisiae altered macrophage inflammasome activity, histone modifications and the gut microbiota in DSS colitis (71, 72). These multilevel effects may be therapeutically relevant, but they do not identify paired modification of NLRP3 or a gasdermin as the active mechanism.

Repurposed and multicomponent interventions have also reduced inflammatory or pyroptotic readouts in rodent colitis. Remdesivir was linked to the SIRT6–FoxC1 pathway, and Xianglian pill affected Toll-like receptor 4–MyD88–nuclear factor kappa B signalling (73, 74). Because these studies involve upstream pleiotropy and preclinical dosing, they support therapeutic context rather than post-translational-modification-specific efficacy.

A butyrate-modified liposome carrying 4-octyl-itaconate accumulated in inflamed colonic epithelium after intravenous administration and reduced GSDME-associated pyroptosis in experimental colitis (75). Human biopsy observations supported disease relevance; treatment efficacy was preclinical. This illustrates delivery to an intestinal compartment, but neither GSDMD inhibition nor dual-modification target engagement.

Further validation should establish acetylation and ubiquitination on the same substrate in defined intestinal cell types, with human tissue observations interpreted alongside functional evidence. Differences in anatomical site, inflammatory activity, medication and cellular composition need to be considered before a tissue modification signal is attributed to UC. Lysine-to-arginine substitutions remove an acceptor for both modifications, and lysine-to-glutamine is an imperfect acetylation mimic; neither alone identifies the responsible modification. Complementary evidence is needed to distinguish coupling from parallel effects on abundance or cell viability.

For translation, a candidate modification state must be linked to an appropriate endpoint. A disease-associated marker is not necessarily predictive of treatment response, and a pharmacodynamic change does not establish clinical benefit. Reduced cytokine release or gasdermin cleavage should be interpreted alongside membrane injury, epithelial repair and host defence. The long-term effects of broad deacetylase or ubiquitin-pathway modulation cannot be inferred from acute colitis experiments. The reviewed studies do not validate a UC biomarker or therapy based on these coupled pathways.

## 7 Conclusions

Acetylation–ubiquitination coupling has experimental support in defined non-intestinal PHGDH–sirtuin, GITR–MARCH7–SIRT2 and HDAC4–GSDMD systems. Most other findings represent separate modifications affecting substrate abundance, activation competence, localisation or persistence. Selected NLRP3 ubiquitin pathways and GSDMD acetylation mechanisms have intestinal relevance, but their convergence is not a complete mechanism validated in human UC. The substrate-state framework helps distinguish these relationships and identify which links remain to be established in intestinal cells.
