# Supplementary material for: Acetylation–ubiquitination crosstalk in NLRP3–gasdermin pyroptosis: a substrate-state framework with relevance to ulcerative colitis
Source: Front Immunol. 2026 Oct 6;17:1976894. doi: 10.3389/fimmu.2026.1976894 (PMC13681192; doi:10.3389/fimmu.2026.1976894)
Supplement: Supplementary file 1 [file Table1.docx]

**Supplementary Table S1**

**Reproducible PubMed search strategy and evidence-audit matrix**

This supplement records the reproducible retrieval strategy and the evidence provenance for representative mechanisms summarised in Table 1 of the main manuscript. It accompanies a targeted narrative review and should not be interpreted as a PRISMA-style systematic-review screening record. Directness (D) and ulcerative-colitis proximity (U) are author-defined descriptive categories, not validated ratings of study quality.

**S1A. Reproducible PubMed search strategy**

| **Run item** | **Recorded value** |
| --- | --- |
| Database and interface | PubMed queried through NCBI E-utilities |
| Reproduction run date | 22 August 2026 (Asia/Shanghai) |
| Eligible publication-date cutoff | 10 August 2026 |
| Date field | 1900/01/01:2026/08/10[dp] |
| Review design | Targeted narrative/mechanistic review; not registered as a systematic review |
| Deduplicated retrieval universe | 14,825 PubMed records across layers A, B, C, E, F and G; layer D is a sensitivity subset |

**Search concept blocks**

| **Block** | **Exact field-tagged terms** |
| --- | --- |
| CORE | (pyroptosis[tiab] OR inflammasome*[tiab] OR NLRP3[tiab] OR gasdermin*[tiab] OR GSDMD[tiab] OR GSDME[tiab]) |
| DISEASE | ("ulcerative colitis"[tiab] OR "inflammatory bowel disease"[tiab] OR colitis[tiab]) |
| ACET | (acetylation[tiab] OR deacetylation[tiab] OR acetyltransferase*[tiab] OR deacetylase*[tiab] OR sirtuin*[tiab] OR SIRT*[tiab] OR HDAC*[tiab] OR KAT*[tiab]) |
| UBIQ | (ubiquitination[tiab] OR ubiquitylation[tiab] OR ubiquitin*[tiab] OR "E3 ligase"[tiab] OR "E3 ligases"[tiab] OR deubiquitinase*[tiab] OR deubiquitination[tiab] OR deubiquitylation[tiab] OR DUB[tiab] OR DUBs[tiab] OR "selective autophagy"[tiab]) |
| DATE | 1900/01/01:2026/08/10[dp] |

**Exact query layers and reproduced hit counts**

| **Layer** | **Purpose** | **Exact PubMed query** | **Hits** |
| --- | --- | --- | --- |
| A | UC/IBD–pyroptosis bridge | ("ulcerative colitis"[tiab] OR "inflammatory bowel disease"[tiab] OR colitis[tiab]) AND (pyroptosis[tiab] OR inflammasome*[tiab] OR NLRP3[tiab] OR gasdermin*[tiab] OR GSDMD[tiab] OR GSDME[tiab]) AND 1900/01/01:2026/08/10[dp] | 1,578 |
| B | Acetylation mechanisms | (pyroptosis[tiab] OR inflammasome*[tiab] OR NLRP3[tiab] OR gasdermin*[tiab] OR GSDMD[tiab] OR GSDME[tiab]) AND (acetylation[tiab] OR deacetylation[tiab] OR acetyltransferase*[tiab] OR deacetylase*[tiab] OR sirtuin*[tiab] OR SIRT*[tiab] OR HDAC*[tiab] OR KAT*[tiab]) AND 1900/01/01:2026/08/10[dp] | 1,431 |
| C | Ubiquitination mechanisms | (pyroptosis[tiab] OR inflammasome*[tiab] OR NLRP3[tiab] OR gasdermin*[tiab] OR GSDMD[tiab] OR GSDME[tiab]) AND (ubiquitination[tiab] OR ubiquitylation[tiab] OR ubiquitin*[tiab] OR "E3 ligase"[tiab] OR "E3 ligases"[tiab] OR deubiquitinase*[tiab] OR deubiquitination[tiab] OR deubiquitylation[tiab] OR DUB[tiab] OR DUBs[tiab] OR "selective autophagy"[tiab]) AND 1900/01/01:2026/08/10[dp] | 1,031 |
| D | Direct crosstalk sensitivity subset | (pyroptosis[tiab] OR inflammasome*[tiab] OR NLRP3[tiab] OR gasdermin*[tiab] OR GSDMD[tiab] OR GSDME[tiab]) AND (acetylation[tiab] OR deacetylation[tiab] OR acetyltransferase*[tiab] OR deacetylase*[tiab] OR sirtuin*[tiab] OR SIRT*[tiab] OR HDAC*[tiab] OR KAT*[tiab]) AND (ubiquitination[tiab] OR ubiquitylation[tiab] OR ubiquitin*[tiab] OR "E3 ligase"[tiab] OR "E3 ligases"[tiab] OR deubiquitinase*[tiab] OR deubiquitination[tiab] OR deubiquitylation[tiab] OR DUB[tiab] OR DUBs[tiab] OR "selective autophagy"[tiab]) AND 1900/01/01:2026/08/10[dp] | 58 |
| E | UC/IBD–PTM bridge without mandatory pyroptosis terminology | ("ulcerative colitis"[tiab] OR "inflammatory bowel disease"[tiab] OR colitis[tiab]) AND ((acetylation[tiab] OR deacetylation[tiab] OR acetyltransferase*[tiab] OR deacetylase*[tiab] OR sirtuin*[tiab] OR SIRT*[tiab] OR HDAC*[tiab] OR KAT*[tiab]) OR (ubiquitination[tiab] OR ubiquitylation[tiab] OR ubiquitin*[tiab] OR "E3 ligase"[tiab] OR "E3 ligases"[tiab] OR deubiquitinase*[tiab] OR deubiquitination[tiab] OR deubiquitylation[tiab] OR DUB[tiab] OR DUBs[tiab] OR "selective autophagy"[tiab])) AND 1900/01/01:2026/08/10[dp] | 1,063 |
| F | Foundational gasdermin/executor mechanisms | (gasdermin*[tiab] OR GSDMD[tiab] OR GSDME[tiab] OR NINJ1[tiab]) AND 1900/01/01:2026/08/10[dp] | 7,475 |
| G | General UC review context | "ulcerative colitis"[ti] AND review[pt] AND 1900/01/01:2026/08/10[dp] | 3,076 |

**Eligibility, prioritisation and citation chaining**

**Eligible evidence.** Primary studies, authoritative mechanistic reviews and clinically relevant UC/IBD studies were retained when they informed a defined pyroptotic checkpoint, an enzyme–substrate relationship, same-substrate acetylation–ubiquitination convergence, or an intestinal/UC bridge mechanism. Priority was given to direct biochemical or genetic evidence, explicit residue or chain-linkage information, perturbation, orthogonal validation and in vivo or human corroboration.

**Exclusions.** Records were not included in the mechanistic synthesis when pyroptosis or the relevant PTM was tangential, when findings could not be assigned to a defined substrate or experimental context, or when an apparent link relied only on generic inflammatory markers. Background reviews were not used alone to assign D1–D3 or U1–U4 categories.

**Citation chaining.** Reference lists of authoritative reviews and sentinel primary studies were checked for foundational work; forward citation checks were used to identify later residue-, enzyme-, linkage- and disease-validation studies. Candidate records whose terminology was absent from title/abstract indexing were rechecked by exact title or DOI. Same-study coupling was distinguished from cross-study convergence.

**Interpretive limitation.** The hit counts define a reproducible retrieval universe; they do not imply that all 14,825 records underwent systematic-review screening. Reliance on one database and relevance-based prioritisation may have omitted eligible studies.

**S1B. Evidence-audit matrix**

D and U are author-defined descriptive categories, not validated ratings of study quality. D1 denotes same-study perturbational support for acetylation–ubiquitination coupling within a substrate or enzyme-regulatory axis; it does not establish same-residue competition. D2 denotes separately tested mechanisms converging on the same substrate, and D3 single-PTM, adjacent-PTM or pathway-level context. U1 denotes cell- or substrate-resolved human UC tissue, U2 patient-derived ex vivo systems, U3 intestinal/colitis models and U4 non-intestinal or reductionist systems. Different findings within one study may have different model provenance. Human expression associations and mixed-IBD cohorts are reported explicitly rather than equated with UC-specific residue-level validation. D1 examples differ in assay and site resolution; the PHGDH study uses combined-site substitutions, whereas the HDAC4 study reports K27 linkage but leaves the ubiquitin acceptor site unresolved. Neither establishes same-residue competition. Acetylation-dependent selection of ubiquitin ligases, readers or routing states remains unproven in UC.

Abbreviations used in the matrix: Ac, acetylation; Ub, ubiquitination; PTM, post-translational modification; UC, ulcerative colitis; IBD, inflammatory bowel disease; NLRP3, NOD-, LRR- and pyrin domain-containing protein 3; NOD, nucleotide-binding oligomerisation domain; LRR, leucine-rich repeat; GSDMD, gasdermin D; GSDME, gasdermin E; ASC, apoptosis-associated speck-like protein containing a caspase activation and recruitment domain; SIRT, sirtuin; KAT5, lysine acetyltransferase 5; HDAC, histone deacetylase; GITR, glucocorticoid-induced tumour necrosis factor receptor; MARCH, membrane-associated RING-CH-type finger; PHGDH, phosphoglycerate dehydrogenase; PP1γ, protein phosphatase 1 gamma; RNF125, ring finger protein 125; TRIM, tripartite motif-containing protein; OTUD6A, OTU deubiquitinase 6A; VANGL2, Van Gogh-like protein 2; OPTN, optineurin; NEK7, NIMA-related kinase 7; NEDD4L, neural precursor cell expressed developmentally down-regulated protein 4-like; USP18, ubiquitin-specific peptidase 18; MIB2, mind bomb E3 ubiquitin protein ligase 2; ERβ, estrogen receptor beta; CPT1A, carnitine palmitoyltransferase 1A; NAD+, oxidised nicotinamide adenine dinucleotide; LPS, lipopolysaccharide; LPC, lysophosphatidylcholine; CLP, caecal ligation and puncture; DSS, dextran sodium sulphate; TNBS, 2,4,6-trinitrobenzenesulphonic acid; AOM, azoxymethane; HEK293T, human embryonic kidney 293T cells; THP-1, human monocytic cell line; DUB, deubiquitinase; IL, interleukin; PMID, PubMed identifier; DOI, digital object identifier. K denotes lysine; K-to-R and K-to-Q substitutions replace lysine with arginine or glutamine.

| **Mechanism / main-manuscript reference** | **Substrate / PTM feature** | **Experimentally demonstrated link** | **Model and checkpoint** | **D** | **U** | **Key limitation and identifier** |
| --- | --- | --- | --- | --- | --- | --- |
| GITR–MARCH7–SIRT2–NLRP3; enzyme-level direct coupling (Ref 36) | NLRP3 K48 ubiquitination/degradation; SIRT2 K48 ubiquitination/degradation and downstream NLRP3 acetylation | GITR competed with NLRP3 for MARCH7, reducing NLRP3 ubiquitination and autolysosomal degradation. Separately, MARCH7 degraded SIRT2, increasing NLRP3 acetylation. | Primary mouse macrophages, HEK293T cells and LPC/CLP/LPS models; human sepsis association | D1 | U4 | Enzyme-level D1 axis outside the intestine; not tested in intestinal models or human UC. PMID 38740925; doi:10.1038/s41423-024-01170-w. |
| PP1γ–HDAC4–GSDMD K248; substrate-level coupling (Ref 44) | GSDMD K248 acetylation and K27-linked ubiquitination; ubiquitin acceptor lysine unresolved | HDAC4-mediated K248 deacetylation reduced K27-linked ubiquitination and pyroptosis. PP1γ diminished HDAC4 inhibition through phosphorylation-dependent regulation. Same-residue competition was not established. | HEK293T and THP-1 cells; LPS-induced mouse inflammation; execution/output | D1 | U4 | K27 linkage was tested; the ubiquitin acceptor lysine and UC relevance remain unresolved. PMID 38326336; doi:10.1038/s41419-024-06505-z. |
| SIRT2–NLRP3 acetylation switch (Ref 7) | SIRT2-dependent NLRP3 deacetylation; functional K21/K22 substitutions | SIRT2 restrained inflammasome activation; single K21R/K22R mutants reduced activation, whereas K24R alone did not show the same inhibitory phenotype. | Macrophages and systemic metabolic inflammation; sensor competence | D3 | U4 | Single-acetylation mechanism; ubiquitination was not causally paired in this study. PMID 32032542; doi:10.1016/j.cmet.2020.01.009. |
| KAT5–NLRP3 mouse K24/human K26 (Ref 8) | Mouse NLRP3 K24 acetylation, corresponding to human K26 | KAT5-dependent acetylation supported oligomerisation. K24R impaired assembly while retaining recruitment to the dispersed trans-Golgi network. | Macrophage/reductionist inflammasome systems; oligomerisation competence | D3 | U4 | Residue-resolved acetylation mechanism; no paired ubiquitination experiment. PMID 38110429; doi:10.1038/s41467-023-44203-0. |
| GSDMD K146 metabolic acetylation (Ref 45) | GSDMD K146 acetylation | D-amino-acid/acetyl-CoA-dependent K146 acetylation reduced GSDMD oligomerisation and macrophage IL-1β release. | Macrophage/reductionist model; execution competence | D3 | U4 | Opposite functional direction to K248 acetylation; ubiquitination was not tested as a coupled arm. doi:10.1126/sciadv.aed1676. |
| RNF125–Cbl-b ubiquitin relay on NLRP3 (Ref 19) | K63- then K48-linked NLRP3 ubiquitination; K496 | RNF125-generated K63 chains enabled Cbl-b recognition and subsequent K48 ubiquitination at K496, promoting proteasomal degradation. | Macrophage/reductionist systems; abundance/termination | D3 | U4 | Sequential ubiquitin editing, not acetylation–ubiquitination coupling. doi:10.1084/jem.20182091. |
| TRIM31–NLRP3 (Ref 12) | K48-linked NLRP3 ubiquitination | TRIM31 directly bound NLRP3 and promoted proteasomal degradation; the study also reported a DSS-colitis phenotype. | Macrophages plus experimental colitis; abundance/disease bridge | D3 | U3 | TRIM31 loss enhanced selected inflammasome responses but ameliorated DSS colitis; no paired acetylation measurement. PMID 27929054; doi:10.1038/ncomms13727. |
| OTUD6A–NLRP3 K430/K689 (Ref 37) | Removal of K48-linked chains from NLRP3 | Myeloid OTUD6A deubiquitinated NLRP3 at K430/K689, increased stability and worsened DSS/TNBS colitis and colitis-associated cancer. | UC transcriptomics and colon tissue (10 UC; 10 cancer-adjacent controls); macrophage/reconstitution and DSS/TNBS/AOM-DSS models | D3 | U3/U4* | Human tissue association is distinct from the experimentally tested site-level mechanism; controls were cancer-adjacent tissue. No paired acetylation. PMID 36932155; doi:10.1038/s41418-023-01148-7. |
| VANGL2–MARCH8–OPTN–NLRP3 K823 (Ref 38) | K27-linked NLRP3 ubiquitination and selective-autophagy routing | VANGL2 recruited MARCH8 to ubiquitinate K823; OPTN recognised the cargo and promoted autophagic clearance, restraining experimental colitis. | Mixed-IBD colon tissue and patient-derived blood-cell experiments; myeloid/reconstitution systems and DSS mice | D3 | U2–U4* | Human observations include UC and Crohn’s disease; they are not UC-only residue-level validation. The ubiquitin/autophagy mechanism was not paired with acetylation. PMID 39899477; doi:10.1371/journal.pbio.3002961. |
| MARCH5–NLRP3 (Ref 39) | K27-linked NLRP3 ubiquitination at K324/K430 | MARCH5-dependent K27 chains supported mitochondrial NLRP3–NEK7 complex formation and inflammasome activation. | Macrophage/non-intestinal systems; localisation/assembly | D3 | U4 | U4 arm of the MARCH5–MARCH8 K27 fate contrast: mitochondrial assembly rather than clearance. Acetylation-dependent selection of this code has not been shown. PMID 37575012; doi:10.15252/embj.2023113481. |
| NEDD4L–GSDMD/GSDME (Ref 47) | GSDMD: K51/K203/K204, K11/K29/K63 linkages. GSDME: K39/K40/K120/K440, K6/K29/K48/K63 linkages. | NEDD4L promoted gasdermin ubiquitination and proteasomal degradation; loss increased gasdermin abundance and cell-death susceptibility with tissue-specific accumulation. | Non-intestinal tissue and cellular models; executioner abundance | D3 | U4 | Intestinal function and acetylation coupling are untested. Read with the published Figure 3 correction. Study doi:10.1038/s41418-025-01598-1; correction doi:10.1038/s41418-025-01653-x. |
| USP18–MIB2–GSDMD K168 (Ref 48) | K33-linked ubiquitination at GSDMD K168 and selective autophagic degradation | USP18 acted independently of its catalytic activity to recruit MIB2, promoting K33-linked GSDMD ubiquitination and selective autophagic degradation. | Macrophage/non-intestinal inflammatory systems; termination | D3 | U4 | Scaffold function, not direct GSDMD deubiquitination; removal of preformed membrane pores and acetylation coupling were not established. PMID 38779488; doi:10.34133/research.0380. |
| NLRP3 K287 neddylation versus TRIM31 (Ref 34) | Neddylation stabilises NLRP3 by opposing degradative ubiquitination | K287 neddylation stabilised NLRP3 and augmented inflammasome-mediated experimental colitis by countering TRIM31-dependent degradation. | Experimental colitis; abundance/persistence | D3 | U3 | Adjacent-PTM interaction, not acetylation–ubiquitination coupling. doi:10.1002/advs.202505906. |
| HDAC6–ubiquitinated inflammasome cargo (Ref 24) | Ubiquitinated-cargo recognition and inflammasome transport by HDAC6 | HDAC6 supported aggresome-like inflammasome assembly; rescue by catalytically inactive HDAC6 supported a scaffold/transport function. | Macrophage/reductionist systems; localisation/termination | D3 | U4 | Enzyme bridge; no same-substrate acetylation–ubiquitination competition. doi:10.1126/science.aas8995. |
| Arctigenin–GSDMD-N K43 acetylation (Ref 46) | Fatty-acid-oxidation-associated GSDMD-N K43 acetylation | ERβ/CPT1A modulation reduced GSDMD-N acetylation and oligomerisation. K43R and K43Q experiments supported K43 involvement; arctigenin attenuated DSS colitis. | THP-1 cells and wild-type/ERβ-deficient DSS mice; macrophage execution | D3 | U3/U4 | K43 was investigated; the acetylation enzyme and coupled ubiquitin route remain undefined. No human UC therapeutic validation. PMID 42390841; doi:10.1021/acs.jafc.6c00666. |
| Composite NLRP3 substrate-state convergence (Refs 7, 8, 12, 37, 38, 39) | Acetylation, degradation, assembly licensing and autophagic routing on NLRP3 | Separate studies converge on NLRP3 abundance, localisation, oligomerisation competence and persistence; no experiment tests interdependence of the acetylation and ubiquitin arms. | Macrophage/reconstitution and colitis models; constituent human UC/mixed-IBD observations | D2 | U2–U4* | Substrate-level synthesis assembled across studies; not a single causal pathway. |
| Composite GSDMD substrate-state convergence (Refs 44, 45, 47, 48; intestinal bridge Ref 46) | K146/K248 acetylation; separate ubiquitin-dependent abundance/clearance; intestinal K43 acetylation | Separate studies regulate GSDMD availability, oligomerisation competence and termination; direct coupling is demonstrated only for the acceptor-unresolved K248–HDAC4 study. | Mixed reductionist, tissue-specific and experimental-colitis systems | D2 overall; Ref 44 is D1 | U3–U4 | Composite evidence must not be described as one universal GSDMD crosstalk axis; Ref 44 is coupling with unresolved acceptor site, not same-residue competition. |
| PHGDH–SIRT1/SIRT3–NLRP3/ASC (Ref 35) | NLRP3/ASC acetylation, ubiquitination and degradation; K21/K22/K24 clusters | PHGDH inhibition/loss increased NAD+ and SIRT1/SIRT3 activity. Combined lysine substitutions and SIRT1 perturbation linked acetylation to ubiquitination and protein stability. | Primary mouse macrophages, HEK293T reconstitution and systemic LPS models | D1 | U4 | Some modification readouts used colocalisation; grouped substitutions do not establish single-site occupancy or competition. No UC validation. doi:10.1016/j.molcel.2024.01.002. |

**Category definitions and abbreviations**

* Human tissue associations are described separately in the model and limitation columns; these do not establish the complete modification mechanism in human UC. D1, direct acetylation–ubiquitination coupling within a defined substrate/site or enzyme-regulatory axis; same-residue competition is a mechanistic subtype, not a requirement. D1 does not imply equal assay resolution or validated evidence quality. D2, convergence of separately tested acetylation and ubiquitination mechanisms on the same substrate. D3, single-PTM, adjacent-PTM or pathway-level bridge evidence. U1, cell- or substrate-resolved human UC tissue; U2, patient-derived ex vivo cells or organoids; U3, experimental intestinal or colitis model; U4, non-intestinal or reductionist system. PHGDH, phosphoglycerate dehydrogenase; PP1γ, protein phosphatase 1 gamma; CLP, caecal ligation and puncture; DUB, deubiquitinase; DSS, dextran sodium sulphate; GSDMD, gasdermin D; GSDME, gasdermin E; LPC, lysophosphatidylcholine; NLRP3, NOD-, LRR- and pyrin domain-containing protein 3; OPTN, optineurin; PTM, post-translational modification; TNBS, 2,4,6-trinitrobenzene sulphonic acid; UC, ulcerative colitis.
